# Complement activation profiles in patients with immune checkpoint inhibitor-associated neuromuscular immune-related adverse events

**DOI:** 10.1007/s00415-025-13181-2

**Published:** 2025-06-12

**Authors:** Leonie Müller-Jensen, Nora Möhn, Thomas Skripuletz, Sophia Carl, Janin Thomas, Lea Grote-Levi, Sandra Nay, Philipp Ivanyi, Imke von Wasielewski, Ralf Gutzmer, Carsten Dittmayer, Werner Stenzel, Samuel Knauss, Matthias Endres, Jan D. Lünemann, Wolfgang Boehmerle, Petra Huehnchen

**Affiliations:** 1 Department of Neurology and Experimental Neurology, Charité – Universitätsmedizin Berlin, Corporate Member of Freie Universität Berlin and Humboldt-Universität zu Berlin, Augustenburger Platz 1, 13353 Berlin, Germany; 2 Berlin Institute of Health (BIH) at Charité – Universitätsmedizin Berlin, Charitéplatz 1, 10117 Berlin, Germany; 3 Department of Neurology, Hannover Medical School, 30625 Hannover, Germany; 4 Immune Cooperative Oncology Group, Comprehensive Cancer Center Hannover (ICOG-CCCH), Hannover, Germany; 5 Department of Hematology, Hemostaseology and Oncology, Hannover Medical School, 30625 Hannover, Germany; 6 Department of Dermatology and Allergy, Skin Cancer Center Hannover, Hannover Medical School, 30625 Hannover, Germany; 7 Johannes Wesling Medical Center, Ruhr University Bochum Campus Minden, Minden, Germany; 8 Department of Neuropathology, Charité – Universitätsmedizin Berlin, Corporate Member of Freie Universität Berlin and Humboldt-Universität zu Berlin, Charitéplatz 1, 10117 Berlin, Germany; 9 Department of Neurology and Experimental Neurology, Charité - Universitätsmedizin Berlin, Corporate Member of Freie Universität Berlin and Humboldt-Universität zu Berlin, Charitéplatz 1, 10117 Berlin, Germany; 10 NeuroCure Cluster of Excellence, Charité – Universitätsmedizin Berlin, Corporate Member of Freie Universität Berlin and Humboldt-Universität zu Berlin, Charitéplatz 1, 10117 Berlin, Germany; 11 Center for Stroke Research Berlin, Charité – Universitätsmedizin Berlin, Corporate Member of Freie Universität Berlin and Humboldt-Universität zu Berlin, Charitéplatz 1, 10117 Berlin, Germany; 12 German Center for Neurodegenerative Diseases (DZNE), Partner Site Berlin, Germany; 13 German Center for Cardiovascular Research (DZHK), Partner Site Berlin, Germany; 14 German Center for Mental Health (DZPG), Partner Site Berlin, Germany; 15 Department of Neurology With Institute of Translational Neurology, University and University Hospital of Münster, Münster, Germany

**Keywords:** Immune checkpoint inhibitor, Immune-related adverse events, Neurotoxicity, Complement activation, Myositis, Neuropathy

## Abstract

**Background:**

Immune-related neuropathy (irNeuropathy) and myositis (irMyositis) are the most common neurologic adverse events (irAE-n) associated with immune checkpoint inhibitors. Although case reports suggest benefits of complement inhibitors, the role of complement activation in irAE-n is understudied.

**Methods:**

In a retrospective multicenter study, we enrolled patients with irNeuropathy or irMyositis, cancer controls (CCs), and healthy controls (HCs). Serum levels of 11 complement components were measured using multiplex enzyme-linked immunosorbent assays. Associations with irAE-n severity and outcomes were assessed by Spearman’s correlation. C5b-9-positive complement deposition was analyzed in muscle and nerve specimens from a subset of patients.

**Results:**

Thirty-one irMyositis patients, 25 irNeuropathy patients, 25 CCs, and 17 HCs were included. Complement component levels were elevated in irNeuropathy (C3a, C5a, sC5b-9, C3, Ba, C4a), irMyositis (C3a, Ba), and CCs (C3a, C5a, sC5b-9, Bb, Ba, C4a), compared to HCs. In irMyositis, higher levels of C5a and complement regulators Factor H and I correlated with lower irAE-n severity (*p* = 0.02, rho = −0.45; *p* = < 0.01, rho = −0.56; *p* = < 0.001, rho = −0.67, respectively), and improved outcomes (*p* = 0.03, rho = −0.42; *p* = 0.05, rho = −0.40; *p* = < 0.001, rho = −0.64, respectively). Subtle C5b-9 deposition was detected in all tissue samples but showed non-specific patterns.

**Discussion:**

Systemic complement activation is detectable in cancer patients regardless of irAE-n status, and tissue complement deposition is unspecific. Our findings suggest that complement activation is not a major driver of irAE-n, leaving the therapeutic potential of complement inhibitors uncertain.

**Supplementary Information:**

The online version contains supplementary material available at 10.1007/s00415-025-13181-2.

## Introduction

Immune checkpoint inhibitors (ICI) have transformed cancer therapy, offering substantial survival benefits for patients with a wide range of malignancies [1]. By modulating immune checkpoints, however, ICI may trigger immune-related adverse events (irAE) that can affect multiple organ systems, including the peripheral nervous system and muscle tissues [2–4]. Immune-related neuropathy (irNeuropathy) and immune-related myositis (irMyositis) together account for approximately two-thirds of all neurologic irAEs and carry a high risk of chronic complications, a relapsing–remitting disease course, or even fatal outcomes [5–7]. While many studies have explored the clinical aspects of neuromuscular irAEs (irAE-n) [8–11], our understanding of the immune mechanisms involved is still incomplete.

The complement system, a central component of innate immunity, plays a critical role in inflammatory disorders such as acute inflammatory demyelinating neuropathy (AIDP) and myasthenia gravis (MG) [12–14]—conditions that can be triggered by immune checkpoint blockade [15–17]. Emerging evidence suggests that complement activation may similarly be involved in the pathogenesis of irAE-n. In a recent study of 11 patients with irMyositis and/or irMyocarditis, single-cell RNA sequencing of heart and skeletal muscle biopsies identified a population of myeloid cells with high expression of FcγRIIIa (CD16a), a receptor involved in IgG binding and associated with increased C3a levels [18, 19]. In the same study, C4d complement deposition was detected in muscle specimens from 7 of 8 patients, 4 of whom tested positive for antibodies targeting muscle antigens [18]. Additional insights emerged from recent case studies reporting clinical benefits of complement inhibition with eculizumab in patients with irMyositis and ICI-related MG [20, 21].

Despite these findings, the exact relationship between complement activation and irAE-n is still largely unknown. In fact, immunohistochemical studies have only rarely detected complement deposition in patients with irMyositis [22, 23]. To date, no studies have specifically examined the involvement of the complement system in irNeuropathy. To address this gap, we conducted a multicenter study to investigate potential dysregulation of the complement system in patients undergoing ICI therapy. We compared serum levels of complement components (C3, C4, C1q), activation products (C3a, C4a, C5a, Ba, Bb, sC5b-9), and regulators (Factor I, Factor H) between patients who developed irMyositis or irNeuropathy, ICI-treated cancer patients without irAEs, and healthy controls. Additionally, formation of the terminal complement complex C5b-9 was analyzed in muscle and nerve specimens from a subset of patients.

## Methods

### Study design and patient selection

We consecutively recruited patients with ICI-related irNeuropathy and irMyositis who presented to the neurology departments of Charité Universitätsmedizin Berlin and Hannover Medical School, Germany, between September 2019 and May 2024. Diagnosis of irAE-n was confirmed, and diagnostic accuracy was determined according to the consensus disease definitions published by Guidon et al. [24]. We enrolled patients with definitive and probable irMyositis and irNeuropathy. Cases of possible irAE-n were only included, if the disease course was highly suggestive of an irAE-n or clinical response to glucocorticoids was observed. The severity of irAE-n was classified following the Common Terminology Criteria for Adverse Events (CTCAE) version 5.0, with a score of “1” meaning mild toxicity and a score of “5” meaning fatal outcome. Demographics and clinical characteristics, including age, sex, type of ICI therapy, concurrent cancer treatments, corticosteroid intake, other irAEs, treatment and outcomes of irAE-n, best tumor response, and progression-free survival (PFS) were obtained from patient’s medical records (Supplemental Table S1). C-reactive protein (CRP) levels, measured as part of routine clinical care within ± 7 days of blood collection for complement component analysis, were extracted as a marker of systemic inflammation. Data on anti-neuronal and rheumatologic autoantibodies (auto-abs) from 10 patients of this study cohort have been reported previously [11].

For correlation analysis, irAE-n outcomes were ranked as follows: complete recovery (“1”), relapsing–remitting course (“2”), recovery with residual symptoms (“3”), and fatal outcome (“4”). Complete recovery was defined as full resolution of neurological symptoms and cessation of immunosuppressive therapy without relapse at the last follow-up. A relapsing–remitting course was characterized by recurrence of neurological symptoms after immunosuppressive treatment tapering, with at least partial symptom improvement following dose escalation. Recovery with residual symptoms was described as persistence of some neurological deficits (e.g., numbness, chronic weakness) unresponsive to immunosuppressive therapy, without symptom flare upon dose reduction. Patients who did not receive immunosuppressive treatment and developed chronic sequelae were also included in this group. Fatal outcome referred to death directly related to irAE-n or its complications, such as irMyocarditis. Cancer outcomes were defined as complete remission (“1”), partial remission (“2”), stable disease (“3”), and progressive disease (“4”). Serum samples from patients with irAE-n, collected in the acute disease stage of the adverse event, were compared to samples from two control groups: healthy individuals (n = 17) and cancer patients receiving ICI treatment who did not develop irAEs within six months of treatment initiation or loss to follow-up (n = 22 and n = 2, respectively), matched for age and sex to the extent possible.

### Sample processing and measurement of complement components

Peripheral blood was collected via phlebotomy, and serum was separated by centrifugation at 1200 × g for 10 min. Serum aliquots were stored at − 80 °C, and only samples with no more than one previous freeze–thaw cycle were selected for analysis. Samples were shipped on dry ice under controlled temperature conditions to the University Hospital of Münster, Germany. Upon arrival, they were thawed in a 37 °C water bath, promptly transferred to ice, and processed within 45 min to minimize in vitro complement activation. Serum levels of complement components (C3, C4, C1q), complement activation products (C3a, C4a, C5a, Ba, Bb, sC5b9), and complement regulators (Factor I, Factor H) were measured using multiplex enzyme-linked immunosorbent assays (ELISAs) based on chemiluminescence according to the manufacturer’s recommendations (Quidel, San Diego, CA; cat. number: A900, A917). To minimize inter-plate variability, samples from all study groups were distributed across each assay plate, along with manufacturer-supplied control samples to ensure consistency. To minimize experimental bias, measurements exceeding the upper or lower limits of quantification were excluded from analysis.

### Tissue staining

Tissue samples were collected when muscle or peripheral nerve biopsies were performed as part of the clinical routine. Cryostat sections, 7 μm thick, were stained using an anti-C5b-9 antibody (DAKO/M777; clone aE11 1:100). Biotinylated secondary antibodies were applied, and the reaction product was visualized using a Benchmark XT immunostainer (Ventana Medical Systems, Arizona, USA). Appropriate positive and negative (technical) controls were included as necessary, with the sarcolemma of normal muscle tissue serving as a negative (physiologic) control. For the semi-quantitative scoring of C5b-9 deposition, ten random fields of vision were examined at × 400 magnification with an Olympus BX50 microscope (Ocular WH10X-H/22). Scores were defined as follows: “- “ meaning absence of C5b-9 deposition, “ + ” meaning rare or focal positive staining, “ + + ” meaning patchy or scattered positive staining, and “ + + + ” meaning strong C5b-9 staining. In muscle tissues, distribution of C5b-9 staining was classified into four categories: (1) diffuse complement staining on the sarcolemma, (2) diffuse complement staining on the sarcolemma and capillaries, (3) focal sarcolemmal complement staining in the perifascicular region, and (4) sarcoplasmic complement staining of necrotic myofibers. In peripheral nerve specimens, staining of the endoneurial capillaries was evaluated.

### Statistical analysis

Categorical variables were compared using the Chi-squared test or Fisher-Exact test, as appropriate. Comparisons of continuous variables between patients with irMyositis and irNeuropathy were performed using the Mann–Whitney U test. For comparisons of continuous data across multiple groups, the Kruskal–Wallis test was applied to assess overall differences, followed by Dunn’s post-hoc test with Bonferroni correction. Spearman’s rank correlation analysis was performed to assess associations between complement components, CTCAE grades, irAE-n outcomes, and best tumor response. Results with *p* < 0.05 were considered statistically significant. To quantify uncertainty, bootstrapped 95% confidence intervals (CIs) for mean rank differences were calculated using 10,000 iterations. Comparisons with p-values < 0.05 but 95% confidence intervals including zero—likely due to small sample sizes—were excluded from further interpretation. For binary categorical comparisons, odds ratios (OR) were calculated as a measure of effect size. Statistical analyses were performed using GraphPad prism (version 10) and R Studio (version 2024.09.0 + 375, R version 4.4.1 (2024-06-14)).

## Results

### Baseline characteristics

A total of 31 patients with irMyositis, 25 patients with irNeuropathy, 25 cancer controls (CCs), and 17 healthy controls (HCs) were included in the study. Baseline characteristics are summarized in Table 1.

**Table 1:** Cohort characteristics

Parameters	irMyositis (n = 31)	irNeuropathy (n = 25)	CC (n = 25)	HC (n = 17)
Median age (IQR), y	66 (60–74)	62 (56–72)	71 (61–78)	61 (57–65)
Sex, no. (%)				
Male	21 (68)	19 (76)	17 (68)	8 (47)
Female	10 (32)	6 (24)	8 (32)	9 (53)
Tumor entity, no. (%)				
Skin cancer (MM, MCC)	17 (55)	8 (32)	13 (52)	–
Lung cancer (NSCLC, SCLC)	7 (23)	7 (28)	3 (12)	–
HCC	4 (13)	1 (4)	8 (32)	–
RCC	1 (3)	4 (16)	0 (0)	–
Others^a^	2 (6)	5 (20)	1 (4)	–
Immunotherapy, no. (%)				
PD-1	18 (58)	10 (40)	15 (60)	–
PD-L1	9 (29)	7 (28)	9 (36)	–
PD-1 + CTLA-4	4 (13)	8 (32)	1 (4)	–
Concomitant tumor therapy, no. (%)				
Chemotherapy	5/29 (17)	8/24 (33)^b^	3 (12)	–
Targeted therapy	8/29 (28)	6/24 (25)	8 (32)	–
None	16/29 (55)	12/24 (50)	14 (56)	–
Intake of glucocorticoids at blood collection, no. (%)				–
Yes	15/26 (58)	6/24 (25)	0 (0)	–
No	11/26 (42)	18/24 (75)	25 (100)	
Best overall tumor response, no. (%)				
CR	1/29 (3)	2/24 (8)	2 (8)	–
PR	7/29 (24)	6/24 (25)	7 (28)	–
SD	16/29 (55)	9/24 (38)	13 (52)	–
PD	5/29 (17)	7/24 (29)	3 (12)	–
Progression free survival (IQR), mo^c^	8 (3–19)	9 (4–19)	14 (7–19)	–

CC, cancer controls; CR, complete remission; CTLA-4, cytotoxic T-lymphocyte-associated protein 4; HC, healthy controls; HCC, hepatocellular carcinoma; ICI, immune checkpoint inhibitor; IQR, interquartile range; MCC, Merkel cell carcinoma; MM, malignant melanoma; mo, months; NSCLC, non-small cell lung cancer; PD, progressive disease; PD-(L)1, programmed death (ligand)−1; PR, partial remission; RCC, renal cell carcinoma; SCLC, small cell lung cancer; SD, stable disease; y, years

^a^Other tumor entities included: Basal cell carcinoma (n = 1), biliary tract cancer (n = 1), cancer of unknown primary (CUP, n = 1), esophagogastric junctional adenocarcinoma (n = 1), head and neck squamous cell carcinoma (n = 1), thymic carcinoma (n = 2), and tonsil carcinoma (n = 1)

^b^Two patients received chemotherapy and targeted therapy as concomitant tumor therapy

^c^Data only available for n = 29 patients with irMyositis and n = 24 patients with irNeuropathy

Statistically significant differences were observed in tumor types and treatment regimens: While patients with irNeuropathy had a lower prevalence of hepatocellular carcinoma compared to CCs (*p* = 0.02; OR, 0.09), they were more likely to have received combination therapy with programmed death (PD)−1 and cytotoxic T-lymphocyte-associated protein (CTLA-)4 inhibitors (*p* = 0.02; OR, 11.29).

A detailed comparison between patients with irMyositis and irNeuropathy is outlined in Supplemental Table S1. In brief, irMyositis developed earlier during ICI therapy, occurring after a median of 2 treatment cycles compared to 4 cycles in irNeuropathy cases (*p* = 0.01). Patients with irMyositis experienced more severe adverse events, as indicated by higher CTCAE grades compared to those with irNeuropathy (median grade: 3 vs. 2; *p* = 0.02). Moreover, irMyositis was associated with a higher likelihood of concurrent non-neurological irAEs (*p* = 0.04, OR = 3.83).

Phenotypes of irNeuropathy encompassed demyelinating presentations including AIDP (n = 7, 28%), axonal neuropathies (n = 3, 12%), or mixed axonal-demyelinating forms (n = 1, 4%, Supplemental Table S1). In 7 (28%) and 7 (28%) patients, respectively, sensorimotor and sensory neuropathy without further specification was diagnosed. In 11 of 25 (44%) patients with irNeuropathy, anti-neuronal or rheumatologic auto-abs were tested within the clinical routine. Five patients (45%) tested positive for one or more autoantibodies targeting SOX-1 (n = 2), GD1b ganglioside (n = 1), acetylcholine receptor (AchR, n = 2), skeletal muscle (n = 1), Ku (n = 1), double-stranded DNA (n = 2), PM-Scl-100 (n = 1), rheumatoid factor IgM (n = 2), citrullinated protein (n = 1), and RNA polymerase III (n = 1). Notably, all auto-abs titers were low and often did not correlate with clinical presentation, suggesting unspecific autoimmune reactions.

In patients with irMyositis, 15 (48%) were diagnosed with concomitant irMyocarditis, including two patients with Triple-M syndrome, defined as irMyositis with irMyocarditis and irMG. Intriguingly, one of these patients had preexisting AchR-ab^+^ MG, which was complicated by irMyositis and irMyocarditis following a single cycle of pembrolizumab for thymic cancer. Additional phenotypes included ocular myositis, dermatomyositis, irMyositis with dropped head syndrome and irMyositis plus irMG (n = 1 each). Auto-abs targeting neuromuscular autoantigens were detected in 11 of 24 (46%) tested patients with irMyositis and encompassed low- to high-titer auto-abs against AchR (n = 5), titin (n = 4), skeletal muscle (n = 8), heart muscle (n = 5), low-density lipoprotein receptor-related protein (LRP)−4 (n = 1), ryanodine receptor (n = 2), and Mi2-alpha (n = 1). Similar to patients with irNeuropathy, most patients (8/11, 73%) exhibited multiple distinct auto-abs.

Regarding irAE-n treatment, glucocorticoids were more commonly used in patients with irMyositis (48% vs. 17%; *p* = 0.02; OR, 4.67), whereas a higher proportion of irNeuropathy patients received no immunosuppressive treatment (17% vs. 54%; *p* = 0.008; OR, 0.18). Additionally, ICI discontinuation was significantly more frequent in patients with irMyositis, with 86% ceasing therapy compared to only 46% in patients with irNeuropathy (*p* = 0.003; OR, 7.39). While there were no statistically significant differences in progression-free survival or tumor response rates between the groups, more patients with irMyositis died from irAE-n, although this difference did not reach statistical significance (*p* = 0.06).

### Complement activation profiles in patients with irAE-n

**Fig. 1:**
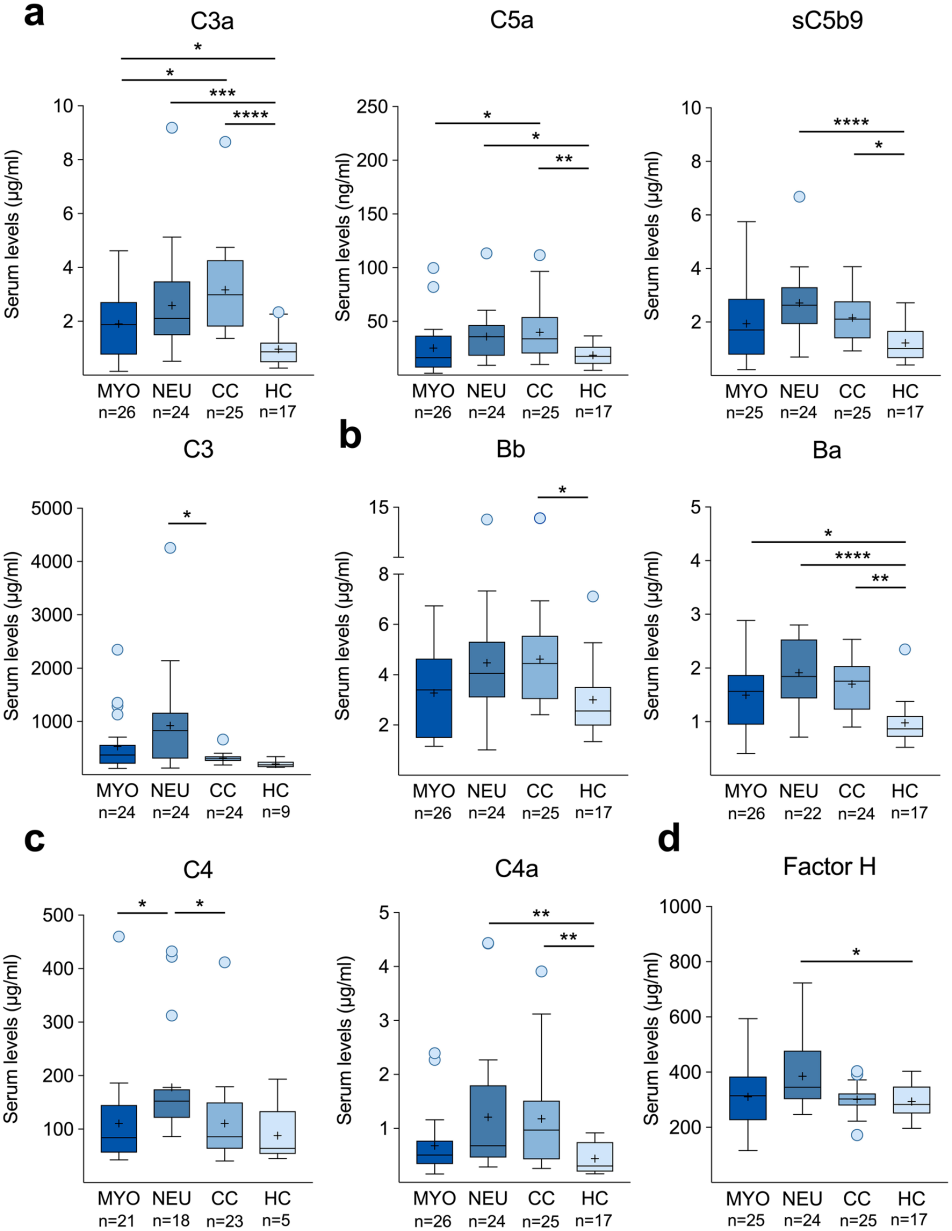
Complement profiles in patients with irAE-n, controls with cancer (CC), and healthy controls (HC). Serum levels of central complement components C3/C3a and components of the terminal complement pathway **(a),** the alternative complement pathway **(b),** the classical and lectin complement pathway **(c)**, and the complement regulator Factor H **(d)** were compared between patients with irMyositis (MYO), patients with irNeuropathy (NEU), CCs and HCs using the Kruskal–Wallis test and the Dunn’s test for post-hoc comparison. Tukey’s box plots display interquartile range (lower and upper edge of the box), range of 1.5 × IQR (whiskers), median (central line), mean (cross), and outliers (dots). * = p < 0.05, ** = p < 0.01, *** = p < 0.001, **** = p < 0.0001

Complement component profiling was completed in 26 of 31 patients with irMyositis (84%), 24 of 25 patients with irNeuropathy (96%), and all controls. First, we assessed activation of central complement components (C3/C3a) and the terminal complement pathway, as indicated by levels of C5a and the soluble form of the membrane attack complex, sC5b-9. Increased serum concentrations were observed in both irAE-n groups and cancer controls (CCs) compared to healthy controls (HCs). Specifically, C3a levels were higher in all three cohorts of cancer patients compared to HCs (irMyositis: *p*_*adj*_ = 0.048; 95% CI, 9.54–33.99; irNeuropathy: *p*_*adj*_ = < 0.001; 95% CI, 20.50–42.73; CCs: *p*_*adj*_ < 0.0001; 95% CI, 34.07–52.50; Fig. 1A), while C3 levels were only increased in patients with irNeuropathy compared to HCs (irNeuropathy: *p*_*adj*_ = 0.04, 95% CI, 3.36–28.73, Fig. 1A). Moreover, C5a and sC5b-9 levels were elevated in irNeuropathy patients and CCs compared to HCs (irNeuropathy: C5a; *p*_*adj*_ = 0.03; 95% CI, 11.05–36.29; sC5b-9; *p*_*adj*_ < 0.0001; 95% CI, 23.40–47.83; CCs: C5a; *p*_*adj*_ = 0.008; 95% CI, 14.16–38.18; sC5b-9; *p*_*adj*_ = 0.01; 95% CI, 12.56–36.52), but not in patients with irMyositis (Fig. 1A).

Exploring the alternative complement pathway, we observed elevated levels of the active subunit of Factor B, Bb, only in CCs compared to HCs (*p*_*adj*_ = 0.03; 95% CI, 9.14—36.50; Fig. 1B). By contrast, levels of the regulatory subunit Ba were increased in all three cohorts of cancer patients—patients with irMyositis (*p*_*adj*_ = 0.03; 95% CI, 9.25 to 35.30), patients with irNeuropathy (*p*_*adj*_ < 0.0001; 95% CI, 28.31 to 52.11), and CCs (*p*_*adj*_ = 0.001; 95% CI, 20.19 to 42.21)—compared to HCs (Fig. 1B).

Lastly, we investigated components of the classical and lectin complement pathway (Fig. 1C) and complement regulators (Fig. 1D). Patients with irNeuropathy presented higher levels of C4 compared to patients with irMyositis (*p*_*adj*_ = 0.01; 95% CI, 3.98 to 30.04) and CCs (*p*_*adj*_ = 0.02; 95% CI, 10.93 to 31.92). In addition, patients with irNeuropathy (*p*_*adj*_ = 0.006; 95% CI, 12.50 to 42.15) and CCs (*p*_*adj*_ = 0.005; 95% CI, 11.80 to 42.33) exhibited increased serum levels of C4a compared to HCs (Fig. 1C). Regarding complement regulators, we observed increased levels of Factor H in patients with irNeuropathy compared to HCs (*p*_*adj*_ = 0.03, 95% CI, 8.11 to 37.56; Fig. 1D). No statistically significant differences in C1q or Factor I concentrations were detected among the four study groups (*data not shown*).

Taken together, our findings indicate that systemic complement activation occurs in ICI-treated cancer patients, irrespective of the presence of irAE-n. Specifically, activation of the alternative pathway and central complement components was observed across all three cancer cohorts, whereas activation of the classical and lectin pathway and the terminal complement pathway was particularly pronounced in patients with irNeuropathy and CCs. Intriguingly, we did not observe C3 or C4 consumption in patients with irAE-n, in contrast to other immune-mediated diseases such as systemic lupus erythematosus (SLE) [25]. Instead, patients with irNeuropathy exhibited notably elevated levels of these complement components.

### Complement activation profiles in auto-ab^+^ and auto-ab^−^ patients with irMyositis

Given the clinical overlap between irMyositis and irMG [16, 26], along with clear evidence of complement activation in patients with idiopathic acetylcholine receptor (AchR) auto-ab positive (AchR-ab^+^) MG [27], we next performed a subgroup analysis comparing serum complement levels between AchR-ab^+^ (n = 4) and AchR-ab^−^ (n = 17) patients with irMyositis. We did not observe significant differences between these two groups (all *p* > 0.05). Interestingly, the patient with preexisting MG exhibited particularly low complement levels, possibly due to being the only patient undergoing plasma exchange at the time of blood collection (Supplemental Figure S1) [28].

Furthermore, we compared complement levels between irMyositis patients with and without *any* neuromuscular auto-ab (n = 9 and n = 12, respectively). Here, we found that C4a levels were *lower* in auto-ab^+^ compared to auto-ab^−^ patients (*p* = 0.04, 95% CI, 0.58 to 9.53), while all other complement component levels remained comparable between the groups (Supplemental Figure S2).

### Complement activation profiles in irMyositis patients and glucocorticoid intake

Compared to patients with irNeuropathy and CCs, complement activation was less pronounced in patients with irMyositis. We hypothesized that this may be attributed to the higher frequency of glucocorticoid use among patients with irMyositis at the time of blood collection (15/26 irMyositis vs. 6/24 irNeuropathy, *p* = 0.02, OR = 4.09; 15/26 irMyositis vs. 0/25 CCs, *p* < 0.0001, OR = ∞; Table 1). However, comparison of patients with irMyositis and glucocorticoid intake and those not receiving glucocorticoids detected no differences in serum levels of complement components (all *p* > 0.05; Supplemental Figure S3).

### Complement activation profiles in patients with demyelinating irNeuropathy

Given the clinical heterogeneity within the irNeuropathy cohort, we hypothesized that complement activation might vary by neuropathy subtype. To test this, we performed a subgroup analysis comparing patients with demyelinating irNeuropathy (n = 7) to those with other irNeuropathy subtypes (n = 17), cancer controls (CCs; n = 25), and healthy controls (HCs; n = 17). No statistically significant differences in complement component levels were observed between irNeuropathy subtypes (all *p* > 0.05; Supplemental Figure S4). Similar to patients with other irNeuropathy phenotypes, those with a demyelinating subtype showed elevated levels of C3a (*p*_*adj*_ = 0.045; 95% CI: 8.34–36.43) and sC5b-9 (*p*_*adj*_ = 0.01; 95% CI: 4.44–44.60) compared to HCs.

### Complement activation profiles and CRP levels

CRP levels are frequently elevated in cancer patients due to infection or the malignancy itself. To further explore systemic inflammation, we compared CRP levels across our cancer cohorts. Median CRP concentrations were 6.4, 6.9, and 4.5 mg/dl in patients with irMyositis, those with irNeuropathy, and CCs, respectively (all *p* > 0.05, Supplemental Figure S5). In the overall cohort of cancer patients, only serum concentrations of Ba and Factor H exhibited a moderate positive correlation with CRP levels (Supplemental Figure S6).

### Correlation between complement components, CTCAE grade, and outcomes

**Fig. 2:**
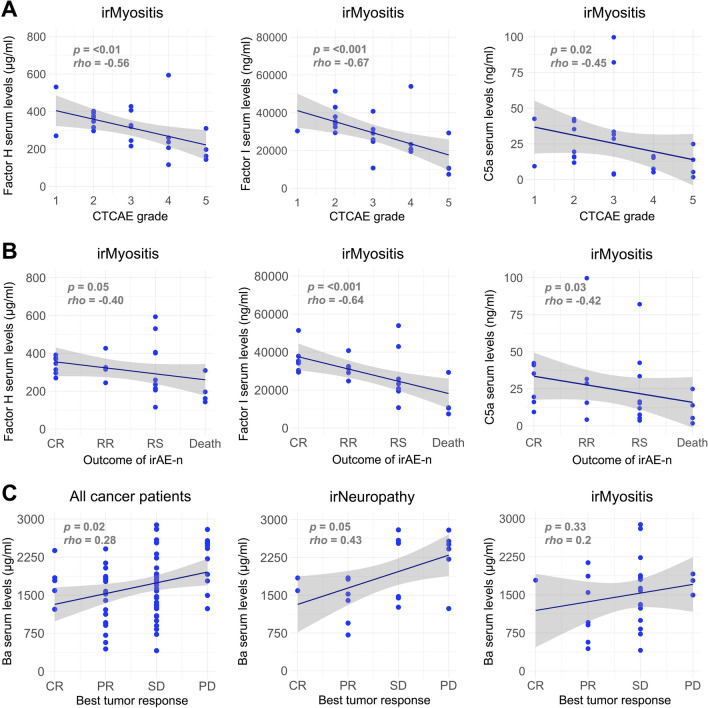
Associations between complement components, irAE-n severity, and outcomes. Serum levels of complement regulators Factor H (upper left) and Factor I (upper middle) as well as serum levels of complement activation product C5a (upper right) show moderate to strong negative correlations with Common Terminology Criteria for Adverse Events (CTCAE) grade, a measure of toxicity severity, in patients with irMyositis (Spearman’s correlation, n = 25, n = 24, n = 26, respectively) **(a)**. Serum levels of complement regulators Factor H (middle left) and Factor I (middle middle) as well as serum levels of complement activation product C5a (middle right) show moderate to strong negative correlations with irAE-n outcomes, defined as complete remission (CR), a relapsing–remitting course (RR), recovery with residual symptoms (RS), or death, in patients with irMyositis (Spearman’s correlation, n = 25, n = 24, n = 26, respectively) **(b)**. Serum levels of complement activation product Ba show a weak positive correlation with tumor outcomes, defined as complete remission (CR), partial remission (PR), stable disease (SD), or progressive disease (PD), in all cancer patients (lower left). In contrast, no statistically significant correlations were observed in patients with irNeuropathy alone (lower middle) and irMyositis alone (lower right,* p* = > 0.05) (Spearman’s correlation, n = 69, n = 19, n = 26, respectively) **(c)**. Each point represents an individual patient. The dark blue line represents the best-fit linear regression line, the grey area depicts the 95% confidence interval for the regression estimate

Serum levels of complement regulators (Factor H, Factor I) and C5a exhibited a moderate to strong negative correlation with both CTCAE grade (a measure of irAE severity, ranging from 1 to 5; Fig. 2A) and irAE-n outcomes (graded 1–4, see Methods and Fig. 2B) in patients with irMyositis. This indicates that higher levels of Factor H, Factor I, and C5a were associated with lower symptom burden and more favorable clinical outcomes. In contrast, no such correlation was observed in patients with irNeuropathy (*data not shown*), potentially due to their lower symptom burden (median CTCAE of 2 vs 3, *p* = 0.02, Supplemental Table S1). Regarding best overall tumor response, Ba levels positively correlated with poor response, meaning that patients with progressive tumor disease exhibited higher Ba levels compared to those who achieved complete remission. This relationship was statistically significant in the overall cohort of cancer patients (*rho* = 0.28; *p* = 0.02; n = 71), but not in patients with irNeuropathy (*rho* = 0.43; *p* = 0.05; n = 21; Fig. 2C) or irMyositis alone (*rho* = 0.2; *p* = 0.33; n = 26).

### C5b-9 deposition in tissue specimens of patients with irAE-n

In the serum, complement activation was detected not only in patients with irAE-n but also in ICI-treated cancer controls. However, serum levels may not necessarily reflect complement activation in the affected tissues. To further investigate this, we analyzed C5b-9 complement deposition in tissue specimens from eight patients with irMyositis, three of whom were also included in the serum complement analysis, and one patient with irNeuropathy. In total, 8 skeletal muscle samples, 3 nerve samples (including two from irMyositis patients), and 1 myocardium sample were examined. Clinical characteristics are summarized in Table 2.

**Table 2:** Characteristics and C5b-9 staining patterns in patients with tissue biopsies

No	Age range	Tumor	ICI	irAE-n	CTCAE	Tissue	Sarcoplasm	Sarcolemma	Capillaries
1	50–59	NSCLC	NIV	MYO	3	skel. muscle (a)	−	+	−
2	60–69	NSCLC	PEM	MYO	na	skel. muscle (b)	−	+	−
3	60–69	Thymic cancer	NIV	MYO	5	skel. muscle (c)	−	+	−
4	60–69	SCLC	ATE	MYO	3	skel. muscle (d)	−	+	+
5	80–89	MM	NIV	MYO	na	skel. muscle (e)	+ +	-	+
6	70–79	MM	PEM	MYO	4	skel. muscle (f)	−	+ *	−
7	50–59	MM	NIV	MYO	2	skel. muscle (g) nerve (i)	−	+ ^(g)^	+ ^(i)^
8	80–89	MM	NIV	MYO	5	skel. muscle (h) nerve (j) myocardium (l)	+ + ^(l)^	+ ^(h)^ −^(l)^	+ ^(j)^
9	70–79	NSCLC	PEM	NEU	na	nerve (k)	na	na	+ +

ATE, atezolizumab; CTCAE, Common Terminology Criteria for Adverse Events; CTLA-4, cytotoxic T-lymphocyte-associated protein 4; ICI, immune checkpoint inhibitor; MM, malignant melanoma; MYO, irMyositis; na, not available; NEU, irNeuropathy; NIV, nivolumab; NSCLC, non-small cell lung cancer; PD-(L)1, programmed death (ligand)−1; PEM, pembrolizumab; SCLC, small cell lung cancer; skel., skeletal

**Fig. 3:**
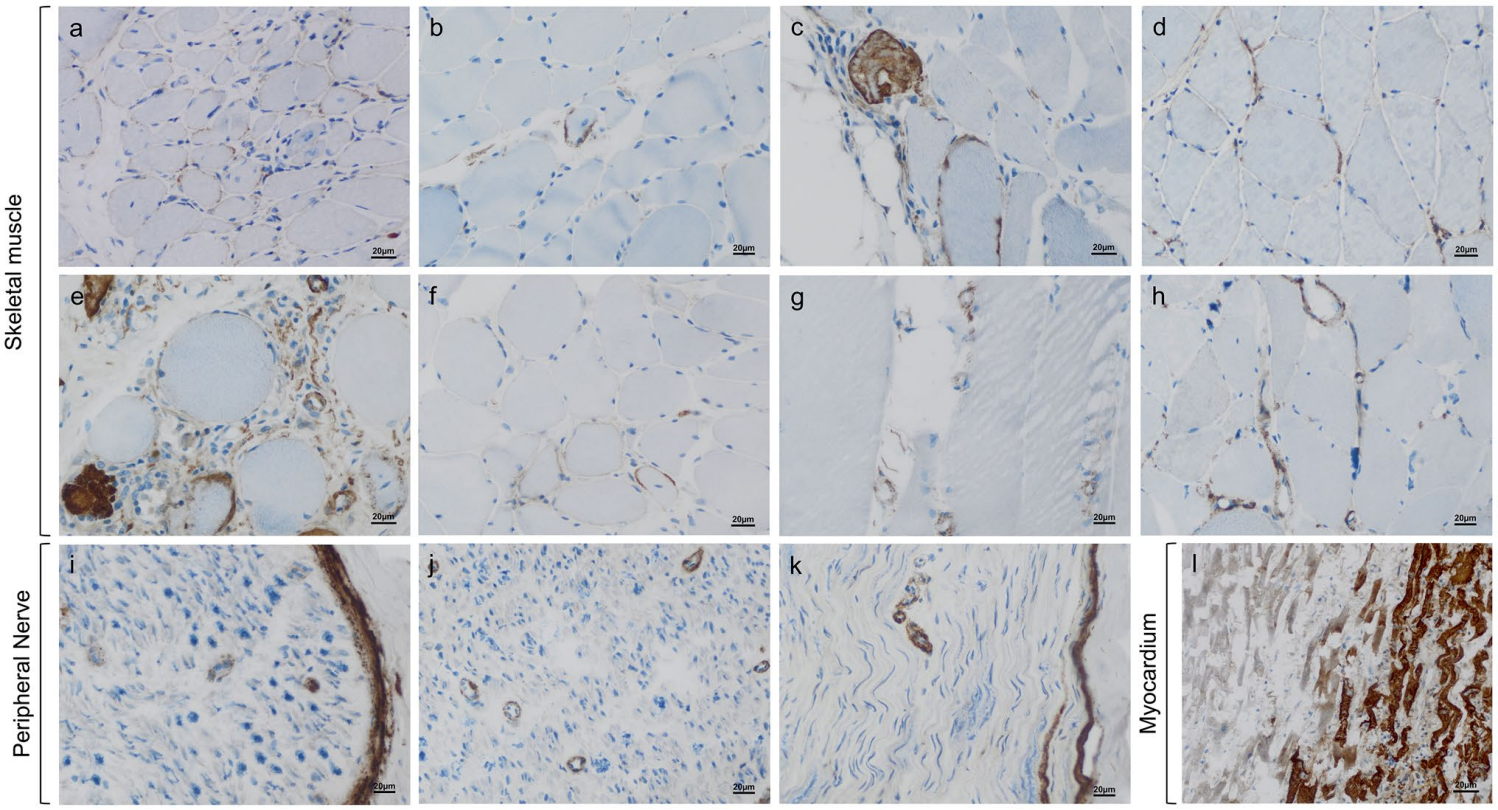
Staining patterns of C5b-9 in patients with irAE-n. Mild sarcolemmal staining of terminal complement complex C5b-9 on non-necrotic myofibers without perifascicular accumulation (**a-c**). Mild C5b-9-positive deposition on the sarcolemma of myofibers and on capillaries (**d**). Sarcoplasmic staining for C5b-9 in necrotic myofibers with complement deposition on thickened capillary walls (**e**). In one patient with irMyositis, sarcolemmal C5b-9 deposition clustered in the perifascicular region (**f**). C5b-9-positive complement deposition on capillaries (**g, h**). C5b-9 deposition on endoneurial capillaries of two patients with irMyositis (**i**, note the physiological perineurial complement positivity, and **j**) and one patient with irNeuropathy (**k**). Myocardial biopsy from a patient with irMyocarditis showing C5b-9 deposition in the sarcoplasm of necrotic cardiomyocytes, while adjacent non-necrotic fibers remain negative (**l**)

Letters (a–k) refer to respective tissue staining in Fig. 3

^*^Sarcolemmal C5b-9 deposits clustered in perifascicular region

In muscle tissue, subtle complement deposition was detected in all samples (Fig. 3A–H). Sarcolemmal C5b-9 staining was present in 7 of 8 specimens (88%) (Fig. 3A–D, F, I); however, only one exhibited C5b-9 clustering in the perifascicular region (Fig. 3F), while all others showed diffusely distributed staining patterns. C5b-9 positivity on individual capillaries was noted in 2 specimens, though without the distinct patterns typically seen in idiopathic myopathies such as in dermatomyositis, anti-synthetase syndrome or immune-mediated necrotizing myopathy (Fig. 3D and 3E) [23]. In 2 samples (25%), non-specific sarcoplasmic C5b-9 staining was detected in necrotic myofibers (skeletal muscle [Fig. 3E] and myocardium [Fig. 3L], respectively), but was absent in non-necrotic fibers. Notably, higher CTCAE scores did not relate to stronger C5b-9 staining patterns (Table 2).

In peripheral nerve tissue, C5b-9 staining was detected on the endoneurial capillary walls of all samples, with additional perineurial staining serving as a physiological positive internal control (Fig. 3I–K). Intriguingly, however, 2 of the 3 patients with available nerve specimens did not exhibit clinical signs of irNeuropathy (Table 2).

In summary, complement deposits were detectable in all muscle and peripheral nerve specimens of patients with irAE-n. However, the degree was generally mild, the distribution highly variable, and the clinical correlation modest at best.

## Discussion

In this multicenter study, we demonstrate that systemic complement activation occurs in ICI-treated cancer patients, independent of the presence of neuromuscular immune-related adverse events (irAE-n). Specifically, we observed activation of all complement pathways in patients with irNeuropathy and controls with cancer, whereas patients with irMyositis primarily exhibited selective activation of the alternative pathway, accompanied by elevated levels of the central complement activation product C3a. To our surprise, higher complement levels correlated with *lower* irAE-n severity, *improved* irAE-n outcomes, and *unfavorable* tumor response in patients with irMyositis. In affected tissues, C5b-9 deposition was consistently detectable, but remained subtle and lacked specific patterns.

Recent case reports have demonstrated clinical efficacy of complement inhibitors in treating ICI-associated irMyositis and Triple-M syndrome [20, 21, 29]. Our findings confirm systemic complement activation in patients with irMyositis, but suggest it results from cancer- or treatment-associated chronic inflammation, as it is equally observed in cancer controls. In the case reported by Nelke et al., muscle biopsy revealed substantial complement deposition on myofibers and capillaries, which provided the rationale for initiating eculizumab treatment [21]. Notably, this patient had preexisting AchR-ab^+^ MG, which was complicated by irMyositis and irMyocarditis after initiating ICI therapy. In idiopathic AchR-ab^+^ MG, serum levels of C3a, C4a, and C5a are reported to be 2- to 3-fold higher than in our irAE-n cohort [27]. Moreover, AchR auto-abs in MG are known to promote complement activation, whereas AchR auto-abs detected in patients with irAE-n may lack effector functions [17]. Consistent with this, neither AchR auto-abs nor other neuromuscular auto-abs were associated with increased serum complement levels in our cohort. Taken together, these findings support a potential role of complement activation in irMyositis secondary to idiopathic AchR-ab⁺ MG, while other irMyositis subtypes may involve alternative immune mechanisms, such as T-cell pathways [22, 30].

Beyond systemic complement activation, autoimmune diseases such as MG, dermatomyositis and AIDP are also characterized by localized complement deposition on skeletal muscles and peripheral nerves [23, 31–33]. In our tissue samples, C5b-9 deposition was common but typically mild, with a variable distribution. Higher CTCAE grades were not linked to stronger complement staining, and C5b-9 deposition was also observed in nerve samples from two irMyositis patients without clinical signs of irNeuropathy, suggesting a weak clinical correlation. Similarly, higher serum levels of complement components were not associated with unfavorable irAE-n outcomes. In fact, increased levels of C5a and the complement regulators Factor H and Factor I correlated with *improved* irAE-n outcomes and *lower* symptom burden in patients with irMyositis. Factor H and I are key regulators that prevent excessive activation of the alternative pathway by inactivating C3b and C4b [34, 35]. Their elevated levels suggest a compensatory response aimed at limiting tissue damage and inflammation. Based on these findings, we believe that complement activation may represent a bystander effect rather than the primary immune mechanism in most irAE-n cases. To support informed treatment decisions, we therefore recommend tissue biopsies for patients with an insufficient response to standard therapies, to distinguish those with substantial complement activation from those without.

In addition to targeted treatment approaches, biomarkers to diagnose and predict irAE-n are currently lacking. In SLE, depleted C3 and C4 levels are widely used as markers of complement consumption and disease activity [36]. In contrast, increased serum concentrations of C3 and C4 have been reported in patients with MS [37]. Moreover, serum levels of C3a and C4a have proven helpful to differentiate immune-mediated diseases from each other, as recently shown for MOGAD, MS, an NMOSD [38]. In our study, patients with irNeuropathy exhibited notably higher C3 and C4 levels compared to CCs and irMyositis patients. However, these levels exhibited high variability and were not associated with irAE-n outcomes. Whether elevation of C3 and C4 reflects a state of cancer-related chronic inflammation or is partially driven by ICI-related autoimmunity therefore warrants further investigation. In this regard, CSF analysis may provide additional insights, as complement activation has been observed in the CSF of patients with idiopathic inflammatory neuropathies such as chronic inflammatory demyelinating polyneuropathy (CIDP) and AIDP [39, 40].

Finally, we found a strong association between unfavorable tumor outcome and elevation of the complement activation factor Ba in patients with cancer. Only recently, Bb levels were associated with poor prognosis in patients with renal cell carcinoma [41]. Given that dysregulated complement activation can foster an oncogenic tumor microenvironment (TME) [42, 43], future studies investigating complement activation specifically within the TME would be desirable.

Our study has limitations. First, the small sample size may have led to an over- or underestimation of effects. Especially, the clinical heterogeneity in the irNeuropathy cohort may have contributed to negative results. However, neuromuscular irAE-n are rare and the largest clinical cohort of patients with irAE-n affecting the peripheral nervous system included 48 patients [6]. Second, while our study included healthy individuals and ICI-treated cancer patients as controls, we were unable to assess complement profiles in cancer patients not receiving ICIs and in patients with non-ICI-related neuromuscular diseases (e.g., idiopathic AIDP). Future prospective studies incorporating these cohorts will be helpful to clarify the effects of ICI therapy on complement activation and to distinguish ICI-related neuromuscular complications from their idiopathic counterparts. Third, 58% of patients with irMyositis and 25% of those with irNeuropathy were receiving glucocorticoids at the time of blood collection. While glucocorticoids do not appear to prevent complement deposition in tissue-based in vitro models [44], it is well established that corticosteroids can reduce complement activity [45] and lower plasma levels of complement components [46]. Although we observed no substantial differences in complement levels between irMyositis patients with or without glucocorticoid therapy, we cannot exclude individual treatment effects. It is therefore possible that complement activation might have been more pronounced in steroid-naïve samples. Fourth, while this study is the first to systematically characterize complement deposition in tissue samples of patients with irMyositis and irNeuropathy, the number of available specimens was limited. To improve our understanding of local immune mechanisms in ICI-associated toxicity, including the role of complement, clinicians should be encouraged to perform muscle and nerve biopsies with a low threshold—provided the potential benefits outweigh risks such as impaired wound healing and infectious complications.

## Conclusion

In summary, our study demonstrates that systemic complement activation is detectable in cancer patients treated with ICIs regardless of the presence of neuromuscular irAEs. Thus, systemic complement activation is not specifically associated with immune checkpoint inhibitor-induced irMyositis or irNeuropathy. Although complement deposition in affected tissues is common, it is typically mild, highly variable in distribution, and shows limited clinical correlation. Based on our findings, complement-mediated inflammation does not appear to be a major driver of irAE-n, leaving the therapeutic benefits of complement inhibitors uncertain. Future prospective studies with larger sample sizes are warranted to validate and expand upon these findings.

## Supplementary Information

Below is the link to the electronic supplementary material.

Supplementary file1 (PDF 2237 KB)

## Data Availability

Anonymized data will be shared by request from any qualified investigator.
