# Supplementary material for: Complement activation profiles in patients with immune checkpoint inhibitor-associated neuromuscular immune-related adverse events
Source: J Neurol. 2025 Jun 12;272(7):459. doi: 10.1007/s00415-025-13181-2 (PMC12162679; doi:10.1007/s00415-025-13181-2)
Supplement: Supplementary file 1 — Supplementary file1 (PDF 2237 KB) [file 415_2025_13181_MOESM1_ESM.pdf]

Complement activation profiles in patients with immune checkpoint inhibitor-associated neuromuscular immune-related adverse events

- Supplemental Material -

Leonie Müller-Jensen, MD, Nora Möhn, MD, Thomas Skripuletz, MD, Sophia Carl, Janin Thomas, Lea Grote-Levi, Sandra Nay, Philipp Ivanyi, MD, Imke von Wasielewski, MD, Ralf Gutzmer, MD[4] Carsten Dittmayer, MD , Werner Stenzel, MD, Samuel Knauss, MD, Matthias Endres, MD, Jan D Lünemann, MD[*], Wolfgang Boehmerle, MD[*], and Petra Huehnchen, MD[*]

[*] These authors contributed equally to this work.

Corresponding author
Dr. med. Leonie Müller-Jensen
Department of Neurology with Experimental Neurology
Charité Campus Virchow
Charité Universitätsmedizin Berlin
Augustenburger Platz 1, 13353 Berlin, Germany
leonie.mueller-jensen@charite.de

Supplemental Table 1: **Clinical characteristics of patients with irNeuropathy and irMyositis.**

**Clinical characteristic**	**irMyositis (n = 31)**	**irNeuropathy (n = 25)**	***p*-value**
**Sex, no. (%), female**	10 (32)	6 (24)	0.56
**Age, median (IQR), yrs**	66 (60-74)	62 (56-72)	0.18
**Tumor entity, no. (%)**			
Skin cancer (MM, MCC)	17 (55)	8 (32)	0.11
Lung cancer (NSCLC, SCLC)	7 (23)	7 (28)	0.76
HCC	4 (13)	1 (4)	0.37
RCC	1 (3)	4 (16)	0.16
Others[a]	2 (6)	5 (20)	0.22
**Immunotherapy, no. (%)**			
PD-1	18 (58)	10 (40)	0.28
PD-L1	9 (29)	7 (28)	>0.99
PD-1 + CTLA-4	4 (13)	8 (32)	0.11
**Concomitant tumor therapy, no. (%)**			
Chemotherapy	5/29 (17)	8/24 (33)[b]	0.21
Targeted therapy	8/29 (28)	6/24 (25)	>0.99
None	16/29 (55)	12/24 (50)	0.79
**Type of irNeuropathy, no. (%)**			
Demyelinating neuropathy / AIDP		7 (28)	
Axonal neuropathy		3 (12)	
Mixed axonal-demyelinating neuropathy	-	1 (4)	-
Unspecified sensorimotor neuropathy		7 (28)	
Unspecified sensory neuropathy		7 (28)	
**Type of irMyositis, no. (%)**			
Myositis with myocarditis	11 (35)		
Ocular myositis with myocarditis	1 (3)		
Dermatomyositis with myocarditis	1 (3)		
Myositis with myasthenia gravis	1 (3)	-	-
Triple-M-Syndrome	2 (6)		
Myositis with dropped-head syndrome	1 (3)		
Unspecified myositis	14 (45)		
**Autoantibodies present, no. (%)**	11/24 (46)	5/11 (45)	>0.99
**CTCAE of irAE-n, median (IQR)[c]**	3 (2-4)	2 (2-3)	**0.02**
**No. of ICI cycles at onset, median (IQR)[c]**	2 (1-4)	4 (3-7)	**0.01**
**Days from irAE-n onset to blood collection, median (IQR)[c]**	18 (9-32)	27 (13-40)	0.44
**Multiple irAE-n, no. (%)[d]**	4/29 (14)	3/24 (13)	>0.99
**Concurrent non-neurological irAEs, no. (%)[e]**	23/29 (79)	12/24 (50)	**0.04**
**Treatment of irAE-n, no. (%)**			
Glucocorticoids	14/29 (48)	4/24 (17)	**0.02**
Glucocorticoids + IVIG	6/29 (21)	5/24 (21)	>0.99
Glucocorticoids + PLEX	1/29 (3)	1/24 (4)	>0.99
IVIG alone	0/29 (0)	1/24 (4)	0.45
Other combinations[f]	3/29 (10)	0/24 (0)	0.24
None	5/29 (17)	13/24 (54)	**0.008**
**Outcome of irAE-n, no. (%)**			
Full recovery	8/29 (28)	6/24 (25)	>0.99
Relapsing-remittent	6/29 (21)	3/24 (13)	0.49
Residual symptoms	10/29 (34)	15/24 (63)	0.06
Fatal	5/29 (17)	0/24 (0)	0.06

Clinical characteristic	irMyositis (n = 31)	irNeuropathy (n = 25)	*p*-value
**ICI stopped due to irAE-n, no. (%)**	25/29 (86)	11/24 (46)	**0.003**
**ICI rechallenge, no. (%)**	6/26 (23)	2/11 (18)	>0.99
**Flare of irAE-n after rechallenge**	4/6 (67)	1/2 (50)	>0.99
**Best overall tumor response, no. (%)**			
CR	1/29 (3)	2/24 (8)	0.58
PR	7/29 (24)	6/24 (25)	>0.99
SD	16/29 (55)	9/24 (38)	0.27
PD	5/29 (17)	7/24 (29)	0.34
**Progression free survival, median (IQR), mo[c]**	8 (3-19)	9 (4-19)	0.89
**Survival at 12 months after irAE-n onset, no. (%)**	16/27 (59)	18/24 (75)	0.37

[a] Other tumor entities included: basal cell carcinoma, biliary tract cancer, cancer of unknown primary (CUP), head and neck squamous cell carcinoma, esophagogastric junctional adenocarcinoma, thymic carcinoma, tonsil carcinoma, (n = 1, respectively). [b] Two patients received chemotherapy plus targeted therapy as concomitant tumor therapy. [c] Data only available for n = 29 patients with irMyositis and n = 24 patients with irNeuropathy. [d] Additional irAE-n included: myasthenia gravis (n = 3) and neuropathy plus encephalitis (n = 1) in patients with irMyositis and encephalitis, hyperCKemia, and neurologic manifestation of Sjögren's syndrome in patients with irNeuropathy (n = 1, respectively). [e] Concurrent non-neurological irAEs included: myocarditis (n = 15), dermatitis or stomatitis (n = 8), thyroiditis (n = 8), arthritis (n = 5), hypophysitis (n = 4), hepatitis (n = 4), colitis (n = 3), pneumonitis (n = 2), gastritis (n = 1), pancreatitis (n = 1), Raynaud's syndrome (n = 1), and vitiligo (n = 1). [f] Other treatment combinations included: glucocorticoids plus mycophenolate mofetil (n = 1), glucocorticoids plus IVIG, PLEX, and eculizumab (n = 1, started after blood collection), glucocorticoids plus cyclophosphamide (n = 1). Statistically significant results ($p = < 0.05$) are shown in bold. Abbreviations: AIDP, acute inflammatory demyelinating polyneuropathy; CR, complete remission; CTLA-4, cytotoxic T-lymphocyte-associated protein 4; HCC, hepatocellular carcinoma; ICI, immune checkpoint inhibitor; IQR, interquartile range; irAE(-n), (neurologic) immune-related adverse event; IVIG, intravenous immunoglobulins; MCC, Merkel cell carcinoma; MM, malignant melanoma; mo, months; MR, mixed response; NSCLC, non-small cell lung cancer; PD, progressive disease; PD-(L)1, programmed death (ligand)-1; PLEX, plasma exchange; PR, partial remission; RCC, renal cell carcinoma; SCLC, small cell lung cancer; SD, stable disease; yrs, years.

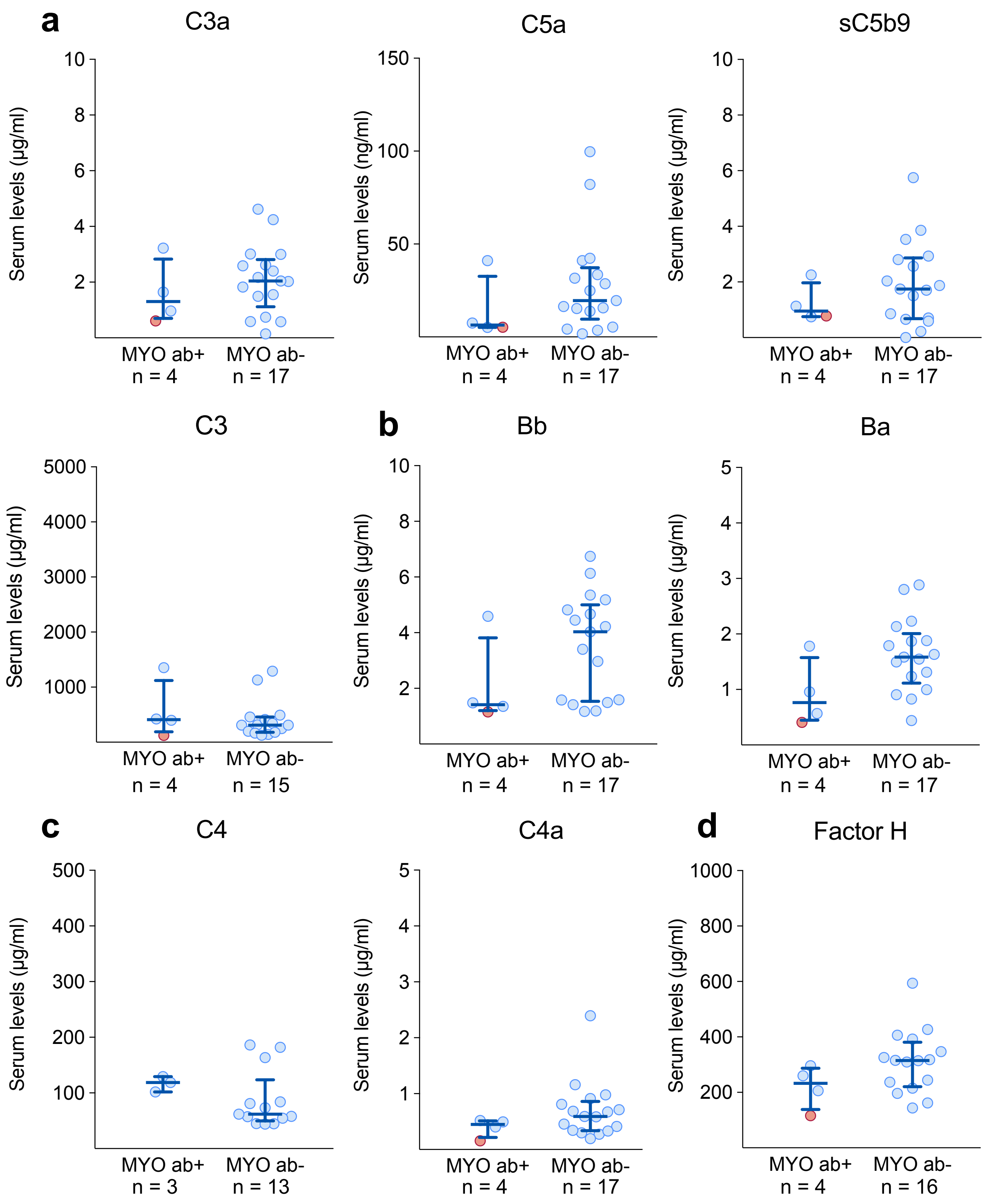

Supplemental Figure 1. Complement profiles in patients with AchR autoantibody-positive irMyositis (MYO ab⁺) and patients with AchR autoantibody-negative irMyositis (MYO ab⁻). Serum levels of central complement components C3/C3a and terminal pathway components **(a),** alternative pathway components **(b),** classical and lectin pathway components **(c),** and complement regulator Factor H **(d)** were compared between MYO ab⁺ (n = 4) and MYO ab⁻ (n = 17) patients using the Mann-Whitney test. No significant differences were observed (all *p*-values > 0.05). Dot plots display individual data points, median, and interquartile range (IQR). The red dot identifies the patient with preexisting AchR ab⁺ myasthenia gravis and secondary irMyositis.

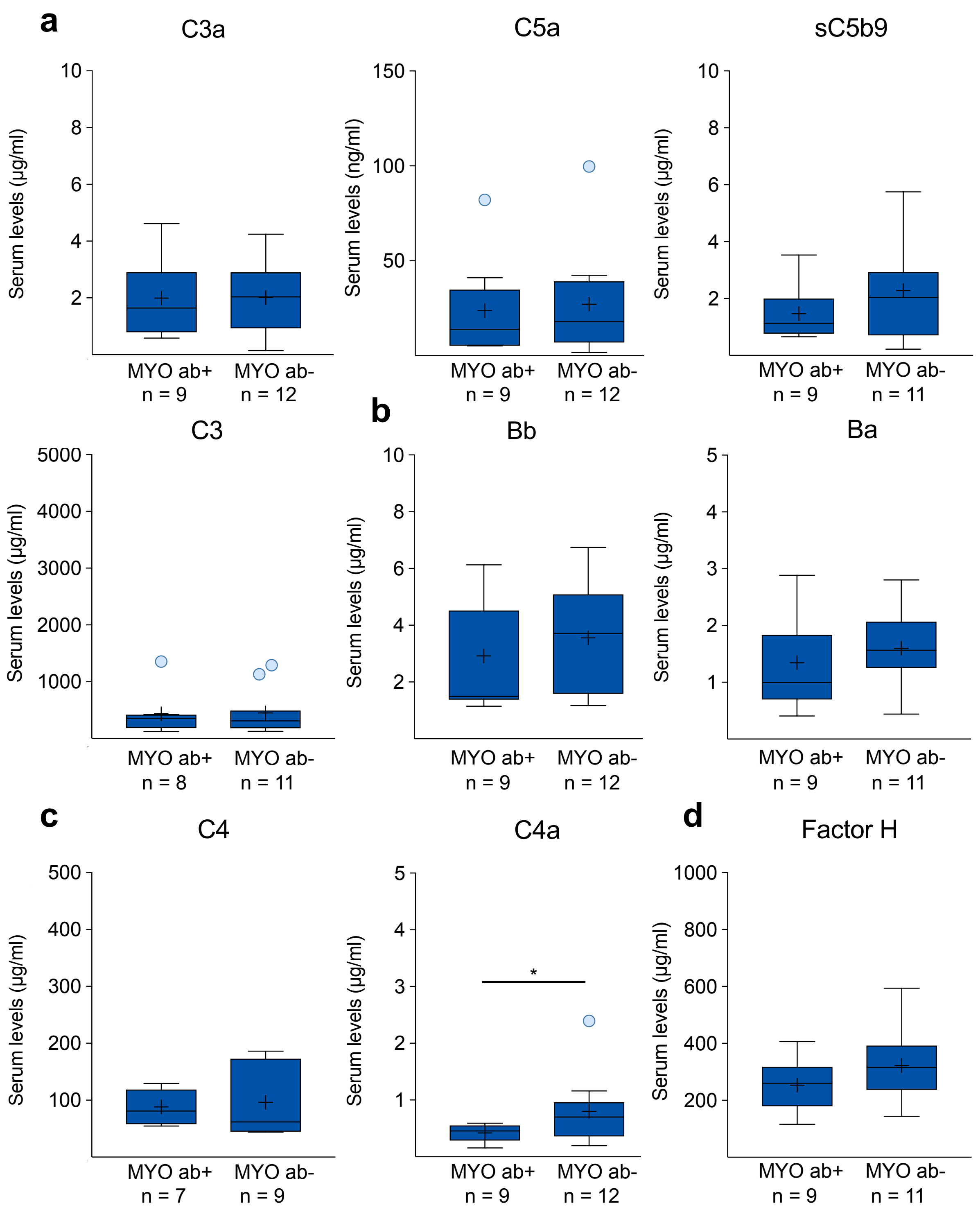

Supplemental Figure 2. Complement profiles in irMyositis patients with (MYO ab⁺) and without (MYO ab⁻) neuromuscular autoantibodies. Serum levels of central complement components C3/C3a and terminal pathway components **(a),** alternative pathway components **(b),** classical and lectin pathway components **(c),** and complement regulator Factor H **(d)** were compared between MYO ab⁺ (n = 9) and MYO ab⁻ (n = 12) patients using the Mann-Whitney test. Only C4a serum levels were higher in MYO ab⁻ patients (p = 0.04), all other *p*-values were > 0.05. Neuromuscular autoantibodies were defined as antibodies targeting titin, skeletal muscle, heart muscle, or AchR. Tukey's box plots display interquartile range (lower and upper edge of the box), range of 1.5 × IQR (whiskers), median (central line), mean (cross), and outliers (dots).

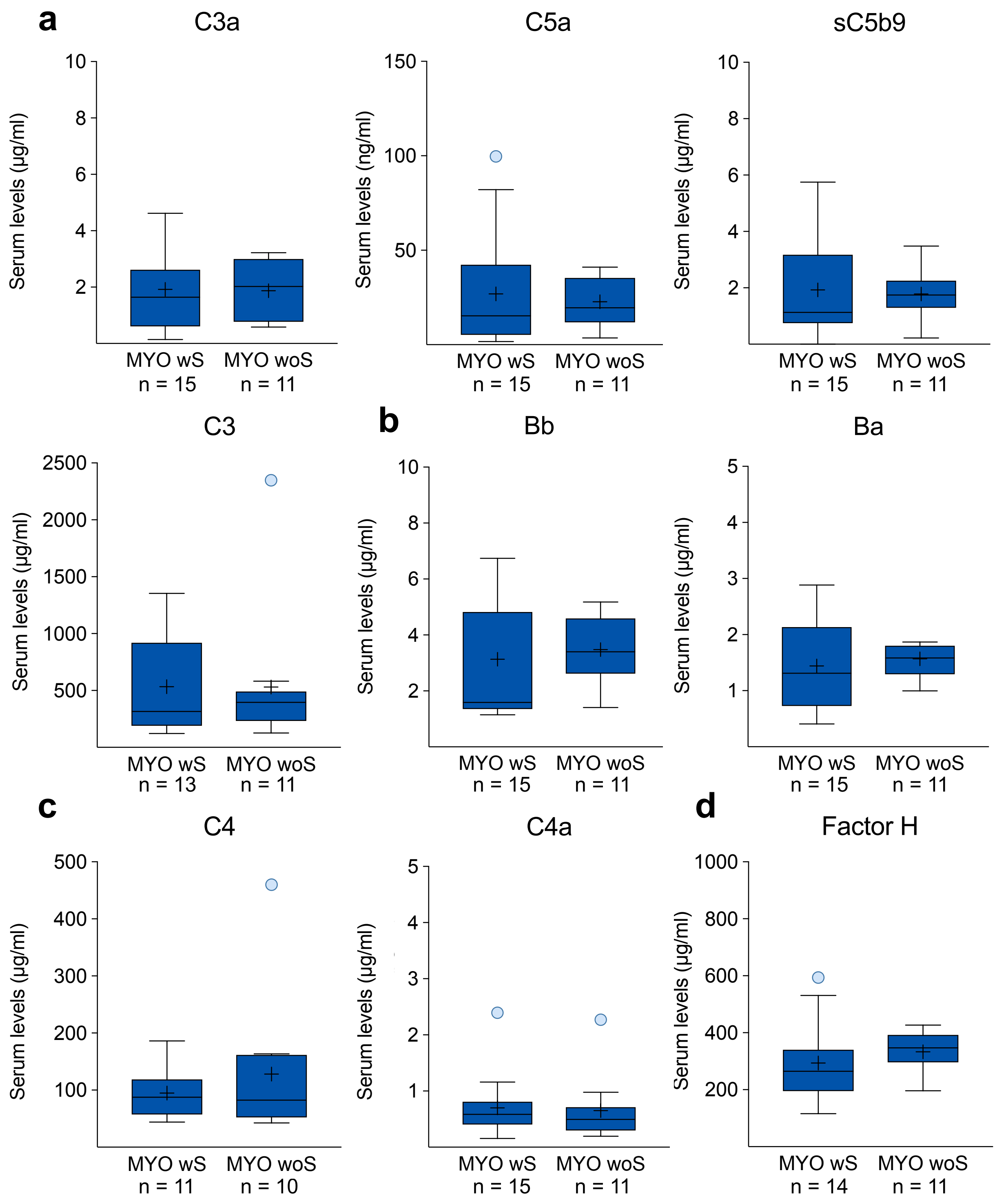

Supplemental Figure 3. Complement profiles in patients with irMyositis receiving glucocorticoids (MYO wS) and patients with irMyositis not receiving glucocorticoids (MYO woS). Serum levels of central complement components C3/C3a and terminal pathway components **(a),** alternative pathway components **(b),** classical and lectin pathway components **(c)**, and complement regulator Factor H **(d)** were compared between MYO wS patients (n = 15) and MYO woS patients (n = 11) using the Mann-Whitney test. No significant differences were observed (all *p*-values > 0.05). Tukey's box plots display interquartile range (lower and upper edge of the box), range of 1.5 × IQR (whiskers), median (central line), mean (cross), and outliers (dots).

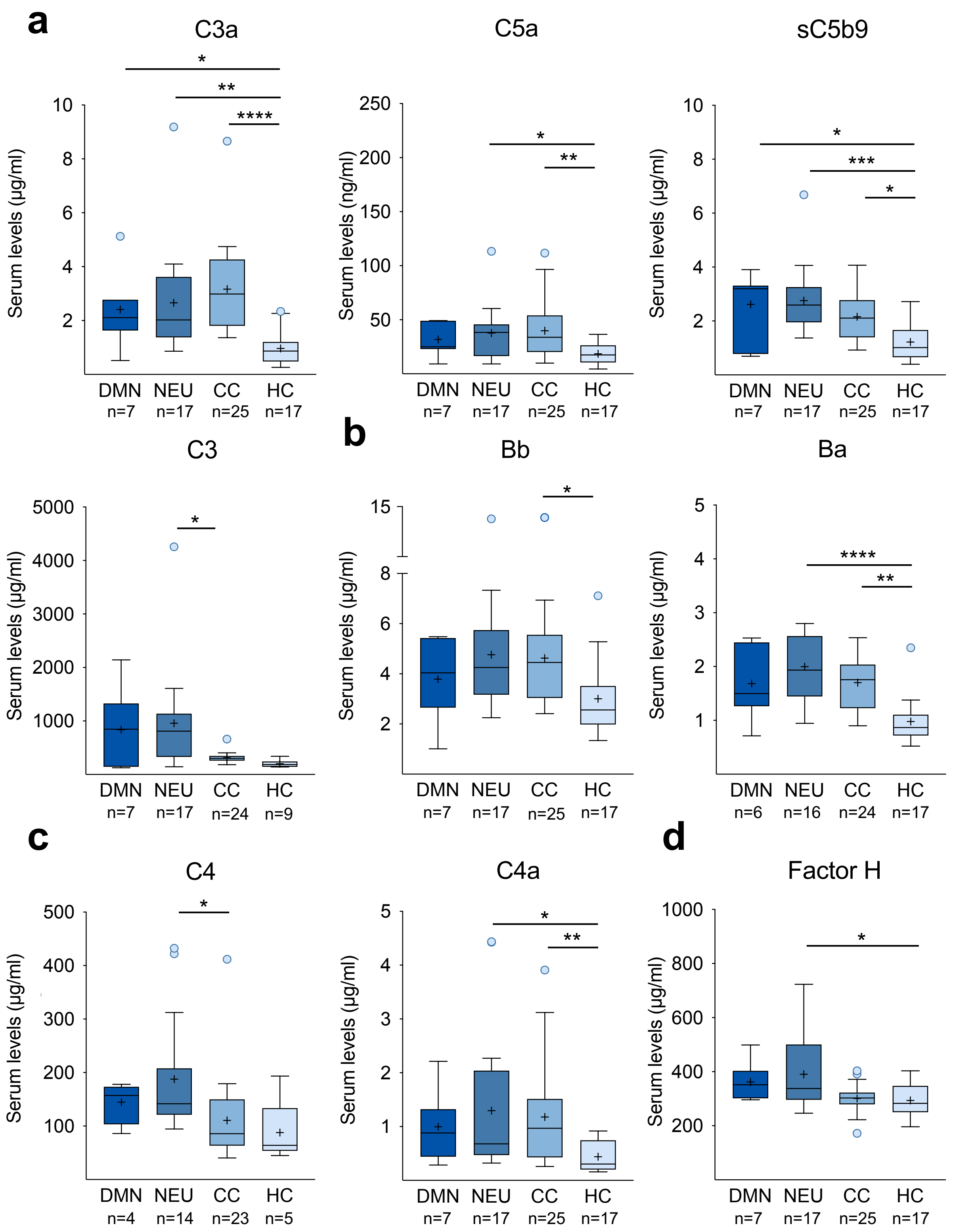

Supplemental Figure 4. Complement profiles in patients with demyelinating irNeuropathy (DMN), patients with other irNeuropathy phenotypes (NEU), controls with cancer (CC), and healthy controls (HC). Serum levels of central complement components C3/C3a and terminal pathway components **(a),** alternative pathway components **(b),** classical and lectin pathway components **(c)**, and complement regulator Factor H **(d)** were compared between DMN (n=7), NEU (n=17), CCs (n=25), and HCs (n=17) using the Kruskal-Wallis test and the Dunn's test for post-hoc comparison. Tukey's box plots display interquartile range (lower and upper edge of the box), range of 1.5 × IQR (whiskers), median (central line), mean (cross), and outliers (dots). * = $p < 0.05$, ** = $p < 0.01$, *** = $p < 0.001$, **** = $p < 0.0001$.

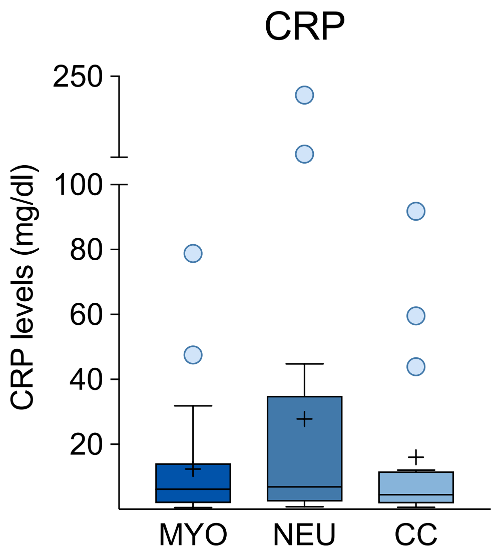

Supplemental Figure 5. Peripheral blood CRP levels in patients with irMyositis (MYO), irNeuropathy (NEU) and cancer patients without irAEs (CC). C-reactive protein (CRP) levels (mg/dl) were compared between MYO (n = 24), NEU (n = 21), and CC (n = 16) using the Kruskal-Wallis test. All *p*-values were > 0.05. Tukey's box plots display interquartile range (lower and upper edge of the box), range of 1.5 × IQR (whiskers), median (central line), mean (cross), and outliers (dots).

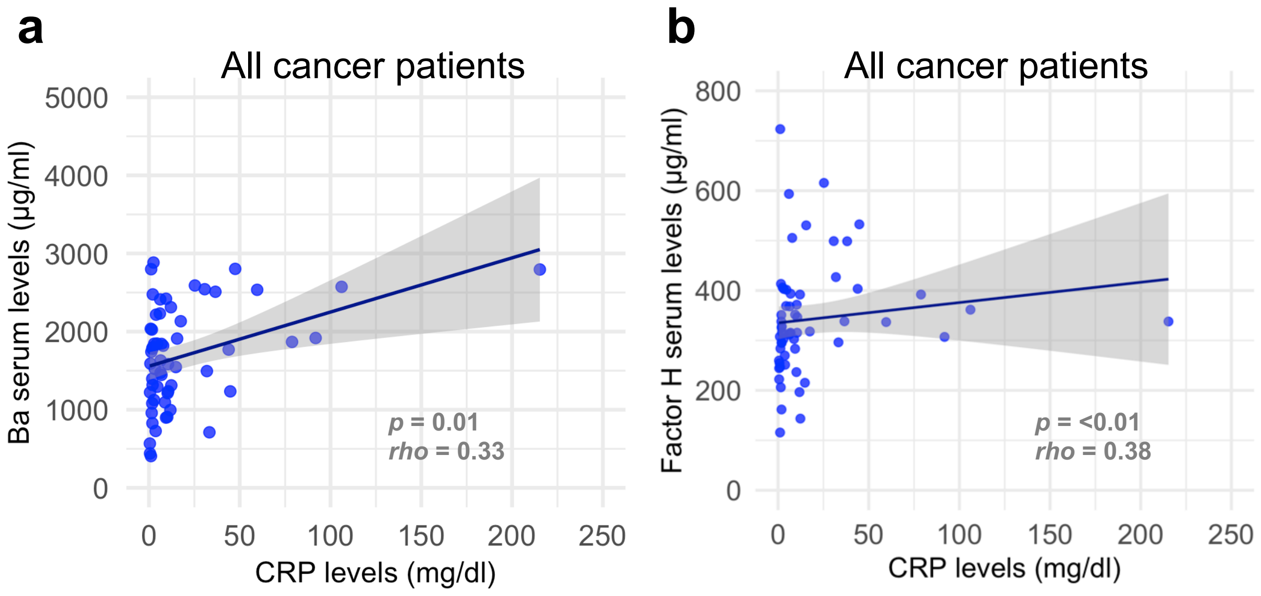

Supplemental Figure 6. Spearman's correlation between peripheral blood CRP levels and complement components in patients with cancer. Serum levels of Ba (µg/ml) show a moderate positive correlation with peripheral blood CRP levels in cancer patients (n = 56; *p* = 0.01, *rho* = 0.33) **(a).** Similarly, serum levels of Factor H (µg/ml) positively correlate with peripheral blood CRP levels (n = 60; *p* = <0.01, *rho* = 0.38) **(b).**
